# Random mutagenesis of *Phaeodactylum tricornutum* using ultraviolet, chemical, and X-ray irradiation demonstrates the need for temporal analysis of phenotype stability

**DOI:** 10.1038/s41598-023-45899-2

**Published:** 2023-12-16

**Authors:** Sean Macdonald Miller, Raffaela M. Abbriano, Andrei Herdean, Richard Banati, Peter J. Ralph, Mathieu Pernice

**Affiliations:** 1 Faculty of Science, Climate Change Cluster (C3), University of Technology Sydney, Sydney, NSW 2007 Australia; 2 Australian Nuclear Science and Technology Organisation (ANSTO), Kirrawee DC, NSW 2232 Australia; 3 Faculty of Medicine and Health, University of Sydney, Camperdown, NSW 2006 Australia

**Keywords:** Environmental biotechnology, Biological physics, Industrial microbiology

## Abstract

We investigated two non-ionising mutagens in the form of ultraviolet radiation (UV) and ethyl methanosulfonate (EMS) and an ionising mutagen (X-ray) as methods to increase fucoxanthin content in the model diatom *Phaeodactylum tricornutum*. We implemented an ultra-high throughput method using fluorescence-activated cell sorting (FACS) and live culture spectral deconvolution for isolation and screening of potential pigment mutants, and assessed phenotype stability by measuring pigment content over 6 months using high-performance liquid chromatography (HPLC) to investigate the viability of long-term mutants. Both UV and EMS resulted in significantly higher fucoxanthin within the 6 month period after treatment, likely as a result of phenotype instability. A maximum fucoxanthin content of 135 ± 10% wild-type found in the EMS strain, a 35% increase. We found mutants generated using all methods underwent reversion to the wild-type phenotype within a 6 month time period. X-ray treatments produced a consistently unstable phenotype even at the maximum treatment of 1000 Grays, while a UV mutant and an EMS mutant reverted to wild-type after 4 months and 6 months, respectively, despite showing previously higher fucoxanthin than wild-type. This work provides new insights into key areas of microalgal biotechnology, by (i) demonstrating the use of an ionising mutagen (X-ray) on a biotechnologically relevant microalga, and by (ii) introducing temporal analysis of mutants which has substantial implications for strain creation and utility for industrial applications.

## Introduction

Humanity faces an uncertain future at the nexus of environmental damage, climate change and insufficient agricultural yield to support a rapidly growing population^1,2^. The challenge lies in balancing both increasing nutrient-rich agricultural yield and preventing environmental damage typically associated with unsustainable farming practices^3^. Microalgae offer a partial solution to this agricultural puzzle by fixing carbon and serving as a direct source of primary and secondary compounds for human consumption^4,5^. In addition, microalgae are being increasingly recognised as renewable and safe sources of a suite of target compounds like carotenoids^6,7^. This is primarily due to the rapid growth and harvesting of microalgae, as well as unique characteristics like the ability to be grown using wastewater and on non-arable land, thereby providing associated advantages like reduced land use^8,9^. However, microalgal biotechnology in general requires research to improve cultivation techniques and strain performance in order to improve economic viability^10^. Advances in genetic manipulation, cultivation technologies, and automation show promise in improving these economic issues^11^.

**Figure 1:**
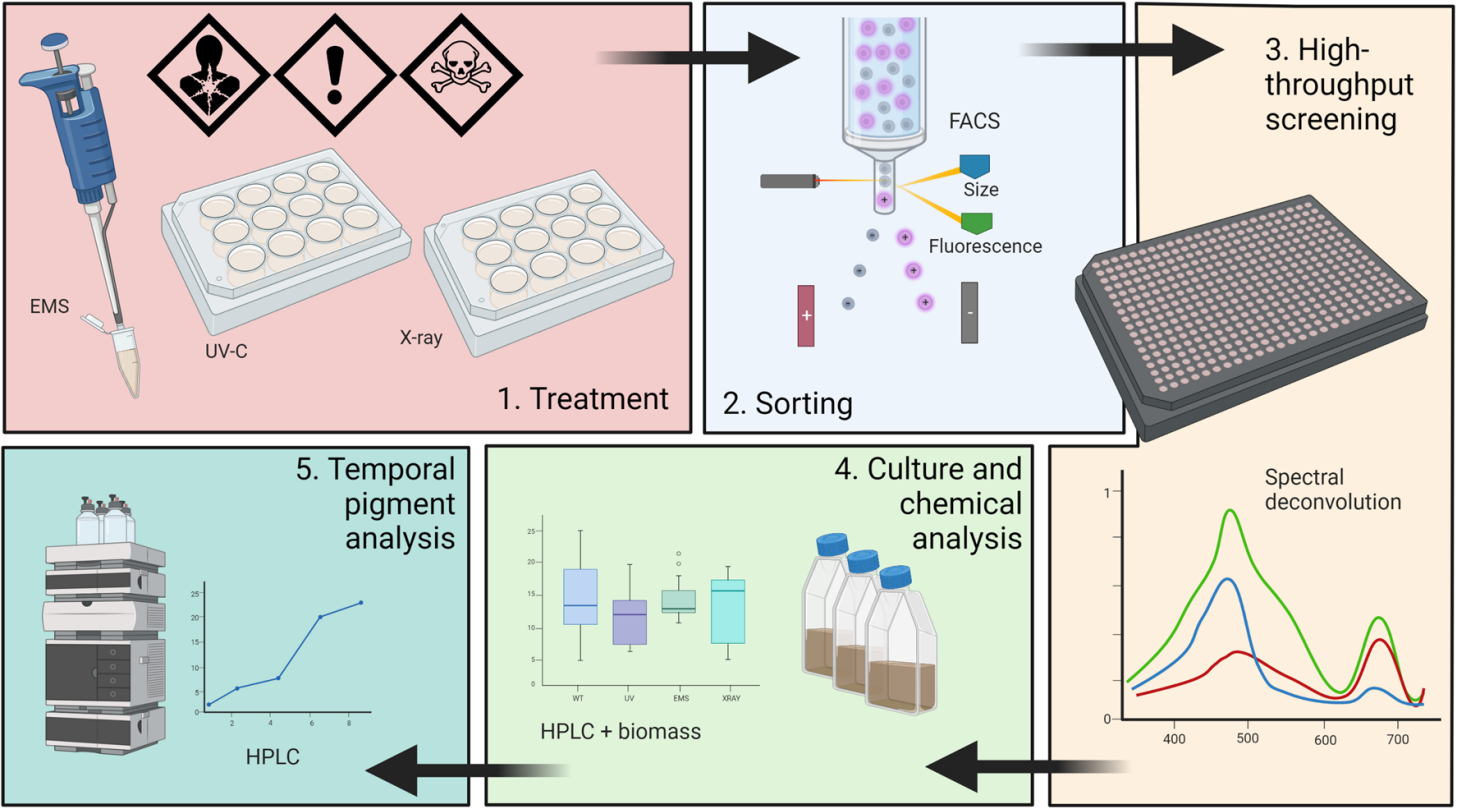
Graphical abstract depicting workflow. (1) treatment using UV, EMS, and X-ray mutagenesis in microtubes and well plates, (2) single-cell sorting into 384-well plates using FACS for size and fluorescence, (3) sterile high-throughput screening using spectral deconvolution with absorbance obtained using plate reader, (4) growth in tissue culture flasks with standard measurements and chemical analysis of pigments using HPLC, and (5) bimonthly pigment analysis using HPLC over a 6-month period.

Genetic engineering targets specific genes to express an enhanced phenotype, however, performing this type of work requires detailed knowledge of microalgal genetics and large infrastructure costs to contain genetically modified organisms. By contrast, experimental or laboratory evolution transcends these challenges by tailoring cells towards improved phenotypes by placing them under a set of conditions in favour of desirable traits^12^. Laboratory evolution can be summarised by the appropriation of two functions of evolution by natural selection—random mutation, which effectively opens new genetic ‘space’ for novel genotypes, and selection pressure, in the form of growth conditions which favour desired phenotypes. A third—artificial selection, provides an expedient means of picking wanted phenotypes (selective breeding). A combination of all three of these is likely to favour successful laboratory evolution towards elite strains. Benefits to an experimental evolution approach include a genome-wide adaptive response to selective pressures rather than targeting specific sites, and the ability to induce desirable changes to genes and regulatory sequences in tandem^13^. Furthermore, unlike genetic engineering, laboratory evolution is not subject to the same restrictive regulatory frameworks for GMO products that present a hurdle for commercialisation. In this work, we investigate two critical components of laboratory evolution—mutagenesis and artificial selection (Fig. 1).

To enable a laboratory evolution approach, mutagens are commonly used as a means to drastically accelerate the natural mutation rate and to generate improved phenotypes for either growth performance or yield of target molecules. Various effects on the genetic material are achievable with different mutagens. In the case of UV mutagenesis, UV-induced molecular lesions are attributable to pyrimidine dimers—cyclobutane pyrimidine dimers and pyrimidine-pyrimidone photoproducts, which in turn account for the majority of genetic effects from UV radiation^14^. EMS is an alkylating agent and alkylates guanine bases, forming O^6^-ethylguanine, resulting in mispairing of guanine bases with thymine instead of cytosine^15^. On the other hand, X-rays have a shorter wavelength than UV rays and are therefore more energetic. As a source of ionising radiation, X-rays produce mutations primarily through double-strand breaks^16,17^. It is likely that differences in the DNA modifications imparted by non-ionising and ionising mutagens will also result in differences in mutant stability, with potential implications on the outcome of laboratory evolution experiments. However, the specific efficacy and stability of each mutagenesis approach is underexplored for biotechnology-relevant microalgal strains.

Random physical and chemical mutagenesis strategies have been employed to create novel algae strains for enhancing lipids, docosaehexanoic and eicosapentanoic acid, as well as for improved CO_2_ fixation and wastewater treatment^18–21^. Random mutagenesis has also been used to improve bioplastic precursors in cyanobacteria^22^. Specific to the current work, UV light and chemically-induced mutagenesis has been used to increase fucoxanthin in *P. tricornutum* up to 170% of wild-type^23,24^. Although there is a lack of recent information on the use of X-ray mutagenesis on microalgae, previous studies on green algae suggest that algal species respond with immense variance to X-ray mutagenesis: Nybom^25^ found 100 Gy to be a lethal dose of X-ray radiation for *Chlamoydomonas reinhardtii*, while Halberstaedter and Back^26^ found *Pandorina morum* could survive up to approximately 300,000 Gy. Kumar used X-ray mutagenesis to create antibiotic resistant strains of *Anacystis nidulans*^27^, though to our knowledge, there are no instances of this mutagen being successfully used to achieve a target of experimental evolution for biotechnological purposes.

Carotenoid compounds play a vital role in photosynthetic organisms where they assist in light harvesting, energy transfer and protection of cellular machinery against oxidation damage^28^. There exists a wide range of health benefits of carotenoids, chiefly as antioxidant molecules, and therefore they are common in the food, feed and pharmaceutical industries^29,30^. The carotenoid fucoxanthin exemplifies these features and is produced with natural yields up to 59.2 mg g^−1^ in the model diatom *P. tricornutum*^31^, and can be enhanced further by artificial selection^32,33^. For these reasons, fucoxanthin production in *P. tricornutum* was targeted in this study.

This work aims to improve understanding of microalgal biotechnology by including a temporal analysis of confirmed positive strains, to investigate phenotype stability in conjunction with proposed best approaches for creating industrial strains. This includes the first examination of ionising (X-ray) mutagenesis as a potential tool for the creation of novel microalgae strains and supports the use of both FACS and the spectral deconvolution method, which enable the exclusion of solvent extraction in high-throughput screening^32^. This information is essential to future experiments aiming to create novel strains using laboratory evolution for biotechnology purposes, in order to both improve screening and selection rates and importantly to maintain strain stability.

## Materials and methods

### Stock culturing

Axenic *P. tricornutum* (CCAP 1055/1) stock cultures were grown in Artificial Sea Water (ASW) medium according to Darley and Volcani^34^ under fluorescent incubator light (150 µmol photons m^−2^ s^−1^) with a 24:0 light cycle in shaking tissue culture flasks (140 rpm) kept at 21 °C.

### Treatment

#### Random mutagenesis

Mutagenesis treatments were conducted by first pipetting 3 mL of each species at 2 × 10^6^ cells mL^−1^ into 1 mL wells in a clear, flat-bottom 48-well microplate (Falcon) and treating without a lid using a 254 nm UV-C Crosslinker (CX-2000, UVP, USA) in triplicate for each treatment time (0, 6, 30 and 300 s). Cell concentration was determined for treatments as well as throughout using flow cytometry (CytoFlex LX, Beckman Coulter, USA) by separating cells into singlets by plotting FSC (forward scatter) against SSC (sidescattter) and using the Cytoflex software statistics functions. For EMS treatments, 1.5 mL of each species was pipetted into 2 mL microtubes and treated with 0.2 M EMS for 0, 30, 60 or 120 min before washing with 5% sodium thiosulfate. X-ray irradiations were performed using an XRAD 320 (PXI, USA) x-ray irradiator. The collimator was removed and the machine was set to its maximum settings of peak energy at 320 kVp and beam current at 12.5 mA. Dosimetry was performed with a calibrated ionisation chamber. Taking into account the attenuation of the plastic well plate covering, dose rates were determined to be 155.3 ± 3.6 mGy/s. This protocol was applied to well plates for multiple doses ranging 4–1000 Gy. A second protocol was applied for lower doses by installing the collimator, reducing the beam current to 10 mA, and increasing the source to sample distance. This dose rate was determined to be 17.9 ± 0.3 mGy/s and applied to doses 1–3 Gy.

All samples were placed in PBS (Phosphate-Buffered Saline) in dark immediately after treatment to limit photorepair of DNA damage^35^. EMS-treated cells were also washed 3× with 5% sodium thiosulfate to inactivate the EMS before resuspension in PBS. After 24 h, the samples were placed in ambient light in 384-well microplates at a final volume of 40 µl PBS for the remainder of the experimental period (7 days). The combination of low light, nutrient depletion and lack of mixing and gas exchange was used to limit cell replication, and in order to reduce the effects of increased salinity from evaporation on mortality, water was pipetted into wells surrounding treated samples while gaps between plate and lids were covered with 2 layers of Parafilm.

#### Mortality assessment

Mortality curves for *P. tricornutum* were established using UV-C and EMS mutagens and a fluorescent cell dye (Invitrogen LIVE/DEAD fixable Violet 405 nm, Thermo Fisher Scientific, USA). Firstly, cell density was chosen based on two factors: a combination of good mutagen penetration into microplates, and sufficient cell quantity for live/dead staining. Two million cells per mL was chosen as this cell density fit both criteria. In order to develop mortality curves, 3 × 0.5 mL of each treatment was dyed at days 1, 3 and 7 after treatment with LIVE/DEAD dye as per the manufacturer’s user guide with slight modification: firstly, the samples were again centrifuged and re-suspended in PBS, then treated with 0.5 µL of LIVE/DEAD dye, vortexed at maximum speed for 5 s (Ratek VM1, Australia) and left in the dark for 30 min. The cells were then washed twice with 0.5 mL of PBS with 1% bovine serum albumin (BSA) obtained from Sigma Aldrich Corp, USA, and re-suspended in 0.5 mL 1% BSA in PBS. Flow cytometry (CytoFlex LX, Beckman Coulter, USA) was undertaken to determine mortality based on dye fluorescence using a 405 nm laser with a 416/451 excitation/emission range. This method was used to retrieve a standard for live positive control cultures using untreated cells. This method was performed identically for another triplicate set of cultures for each species to attain dead negative control culture references, with the exception that these samples were heated in sealed 30 mL glass test tubes to 90 °C+ on a hot plate (Major Science MD-01N, USA) for over 10 min prior to measurement. Complete culture mortality was confirmed by grouping of at least 98% of cells at ~ 1% of the chlorophyll fluorescence of wild-type cultures.

### Sorting and screening

#### Sorting

**Figure 2:**
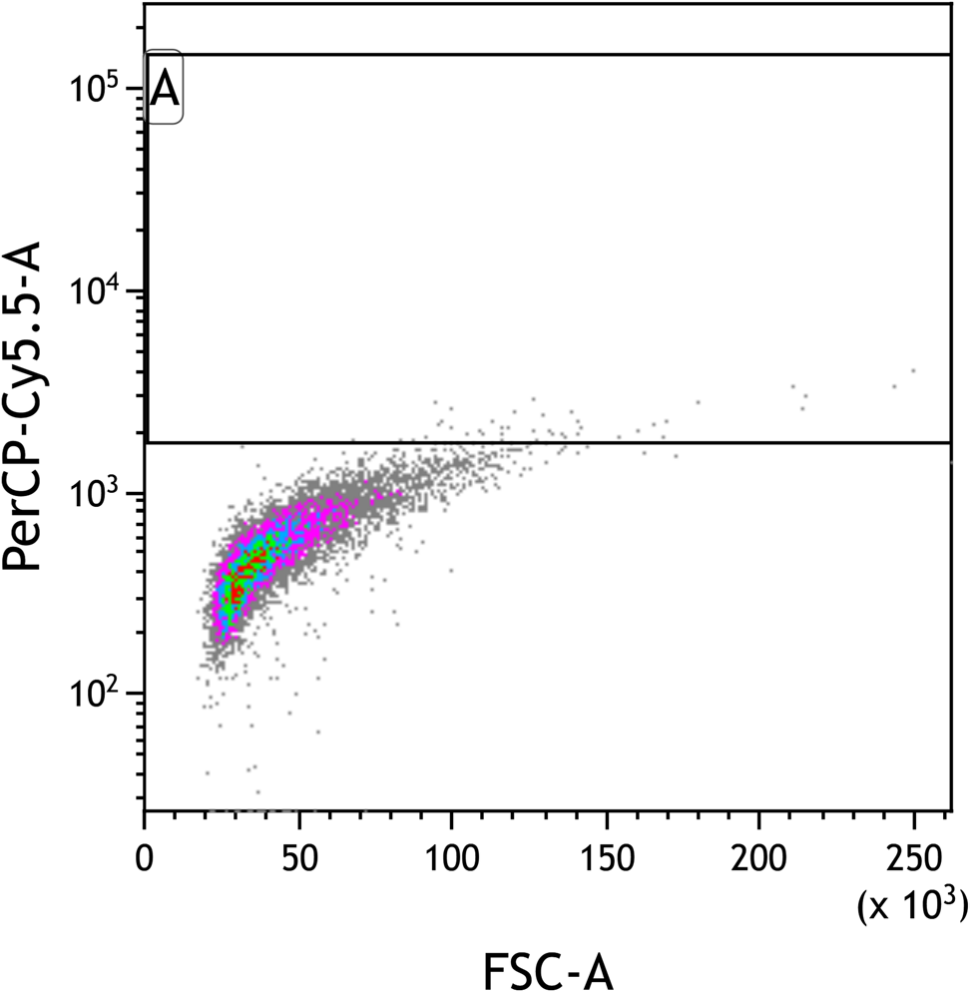
Fluorescence Activated Cell Sorting (FACS) gating strategy. FSC (forward scatter) is plotted on the x-axis against PerCP-Cy5.5.5-A on the y-axis. The latter has previously shown to have high correlation to fucoxanthin content measured using HPLC. The top 1% cells are selected for single-cell sorting (box A).

The treatments chosen for sorting with FACS were 6 s (UV), 30 min (EMS) and 1000 Gy (X-ray) as well as a control using WT which were all single-cell sorted on day 7 after treatment. A straight line gate was used for sorting single cells into 384-well microplates using FACS (BD FACS Melody, Beckman Coulter, USA). Firstly, FSC was plotted against SSC to separate singlets after which treated cultures were sorted by gating for the highest fucoxanthin using the PerCP-Cy5.5 channel (Fig. 2). The ~ 1% highest-fluorescing events were selected for further screening by sorting single cells into black, flat-bottom 384-well microplates in ASW. These cells recovered over a period of ~ 10 days in incrementally higher light intensities: ~ 5, 10, 25, 50 and finally 100 µmol photons m^−2^ s^−1^ white light in a shaking incubator (Climo-Shaker ISF1-XC, Kuhner, Switzerlamd) with a 24:0 light cycle at 95 rpm and 21 °C to reduce damaging effects from excessive light on single cells.

#### Screening

Once surviving wells were between an OD of 0.2 and 1.0 when measured at 750 nm using microplate reader (Infinite M1000 Pro, Tecan, Switzerland), spectra from 400 to 700 nm was measured and used for spectral deconvolution screening, the full method is outlined in our previous work^32^. The top-performing mutants were selected based on both high optical density and spectral deconvolution results. The top-performing strains from each mutagen were measured for biomass and pigment content, after which the top replicate was sub-cultured for 6 months and measured bimonthly for pigment content thereafter.

### Measuring culture characteristics and sampling

One strain from each treatment was identified for further analysis of growth characteristics and pigment content. These strains were cultured in ASW under fluorescent light at 150 µmol photons m^−2^ s^−1^ with a 24:0 light cycle at 50 mL in 250 mL shaking tissue culture flasks (140 rpm) kept at 21 °C. Daily 1:2 dilutions were included to ensure optimal light and nutrient availability for 3 days, after which the flasks were placed in low light (< 10 µmol photons m^−2^ s^−1^) without dilution for an additional 5 days to maximise carotenoid content. Cell counts and chlorophyll fluorescence were measured daily using flow cytometry.

At the end of the experimental period, cultures were centrifuged, washed with MQ and flash-frozen in liquid nitrogen before being lyophilised and stored at − 80 °C for further analysis. Data visualisation was performed using GraphPad Prism version 9.0.2 for Windows (GraphPad Software, San Diego, California USA, http://www.graphpad.com) and Kaluza Flow Cytometry Analysis Software version 2.1 (Beckman Coulter, USA).

Biomass productivity was measured using the equation Productivity = Ln(N_2_/N_1_)/(t_2_ – t_1_) where N is found using absorbance at 750 nm. Fucoxanthin productivity was calculated by multiplying fucoxanthin content by N at any given time point.

### High performance liquid chromatography

Freeze-dried pellets were weighed and re-suspended at 2–3 mg dry biomass per mL of chilled ethanol before being sonicated with 1-s pulses using an ultrasonic homogeniser (Qsonica Q125) at 100% amplitude and filtered once extraction was confirmed. Extracts were filtered using 0.2 µm PTFE syringe filters and stored in − 80 °C until analysis. High Performance Liquid Chromatography (HPLC) was conducted using an Agilent Technologies 1290 Infinity, equipped with a binary pump with an integrated vacuum degasser, thermostatted column compartment modules, Infinity 1290 auto-sampler and PDA detector. Column separation was performed using a 4.6 mm × 150 mm Zorbax Eclipse XDB-C8 reverse-phase column (Agilent Technologies, Inc.) and guard column using a gradient of TBAA (tetrabutyl ammonium acetate): Methanol mix (30:70) (solvent A) and Methanol (Solvent B) as follows: 0–22 min, from 5 to 95% B; 22–29 min, 95% B; 29–31 min, 5% B; 31–40 min, column equilibration with 5% B. Column temperature was maintained at 55 °C. A complete pigment profile from 270 to 700 nm was recorded using PDA detector with 3.4 nm bandwidth.

### Statistical analysis

One-way ANOVA with a confidence level of 0.05 was performed followed by Tukey’s post-hoc test to determine significance among samples, using GraphPad Prism version 9.0.2 for Windows, GraphPad Software, San Diego, California USA, http://www.graphpad.com.

## Results

### Mutagen effects on mortality and chlorophyll fluorescence

**Figure 3:**
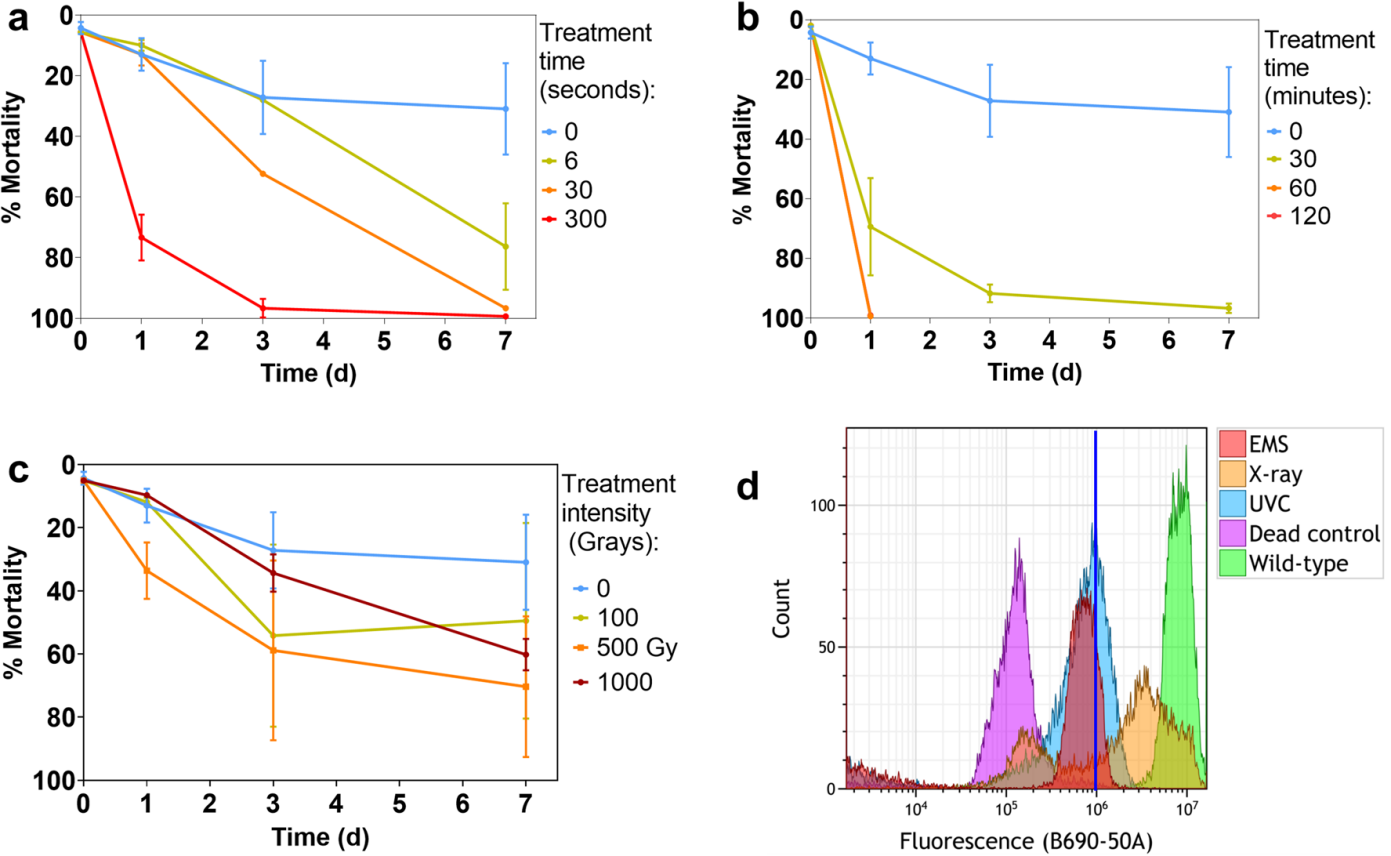
Mortality and fluorescence assessment examples for selected treatments. (**a**) UV-C (Note that data is missing for day 3 6-s treatment), (**b**) EMS and (**c**) X-ray treatments over a 7-day period, *n* = 3. For clarity not all treatments from the mortality assessment are included. (**d**) Chlorophyll fluorescence measured using the flow cytometry B690 channel as proxy at day 3 for treatments from each mutagen used for downstream analysis of mutants (6-s UV, 30-min EMS and 1000 Gy X-ray). The positive control is healthy WT *Phaeodactylum tricornutum* cells while the negative control is the same cells boiled on a hot plate until dead.

Cell death and chlorophyll fluorescence were assessed over a 7-day period in order to gain a preliminary understanding of the effects of UV, EMS and X-ray treatments on *P. tricornutum* cultures. Mortality was high for 6 s treatments of UV (${\overline{\text{x}}}$ = 76 ± 14), 30 s of UV (${\overline{\text{x}}}$ = 97 ± 7) and 300 s (100%) of UV 7 days after treatment (Fig. 3a). At this time point, mean mortality was 97% (± 1.5) for 30-min EMS and 100% for both 60- and 90-min EMS treatments (Fig. 3b) and both UV and EMS mortality were similar to what was previously observed in the literature^23,24^. X-ray mortality was irregular across treatment intensity and displayed high errors between treatments, except 1000 Gy, which resulted in a mean 60 ± 5% mortality (Fig. 3c). Despite the 30-s UV-C treatment having identical mortality as the 30-min EMS treatment (×97%), the slope for 30-s UV-C was still steeply declining at day 7, unlike the 30-min EMS which is nearing asymptote. For this reason, the 30-min EMS treatment was chosen whereas 6-s treatment was chosen instead for UV as the appropriate treatment with day 7 selection point for further analysis. The X-ray treatment chosen for further mutagenesis screening was 1000 Gy as it displayed a combination of high mortality and low error at day 7 (${\overline{\text{x}}}$ = 60%, SD = 4.9%). These treatments were chosen for further analysis because they were generally high and also because based on the shape of the mortality curves it was believed the mortality was likely to continue increasing after day 7 for each treatment. Indeed, mortality increased after single-cell sorting at day 7. The chosen UV treatment exhibited 76% mortality at day 7 after treatment with LIVE/DEAD dye, while the same treatment exhibited 96% mortality after single-cell sorting. The same pattern is seen for the EMS treatment with 97% mortality on day 7 and 99.7% mortality after sorting. This is not true however for X-ray treatments which showed 60% mortality on day 7 and only 11% after single-cell sorting. Whether this mortality after sorting is due to increased stress from the sorting process or from ongoing effects from mutagenesis is not certain.

While EMS and UV treatments showed defined differences in mortality between treatment intensities as well as low error (Fig. 3a and b), X-ray treatments did not show distinct mortality statistics based on treatment intensity (with 500 Gy displaying a higher mean mortality than 1000 Gy) and often displayed high variability (Fig. 3c). In addition, chlorophyll *a* fluorescence declined evenly throughout the 7 days after treatment with UV-C and EMS-treated cultures, whereas X-ray treated samples showed a more complex separation of the fluorescence into two separate populations. This can be seen best on day 3, with one group firmly embedded within the negative control group (dead) and a second towards the positive control group (alive, Fig. 3d).

These results suggest that UV-C and EMS produce more consistent results within a culture, while X-ray asymmetrically affects cell survival and fluorescence. While both UV-C and EMS mutagens produced visibly different trends between treatment intensities, X-ray treatments often overlapped and had high variability.

### Screening and HPLC

**Figure 4:**
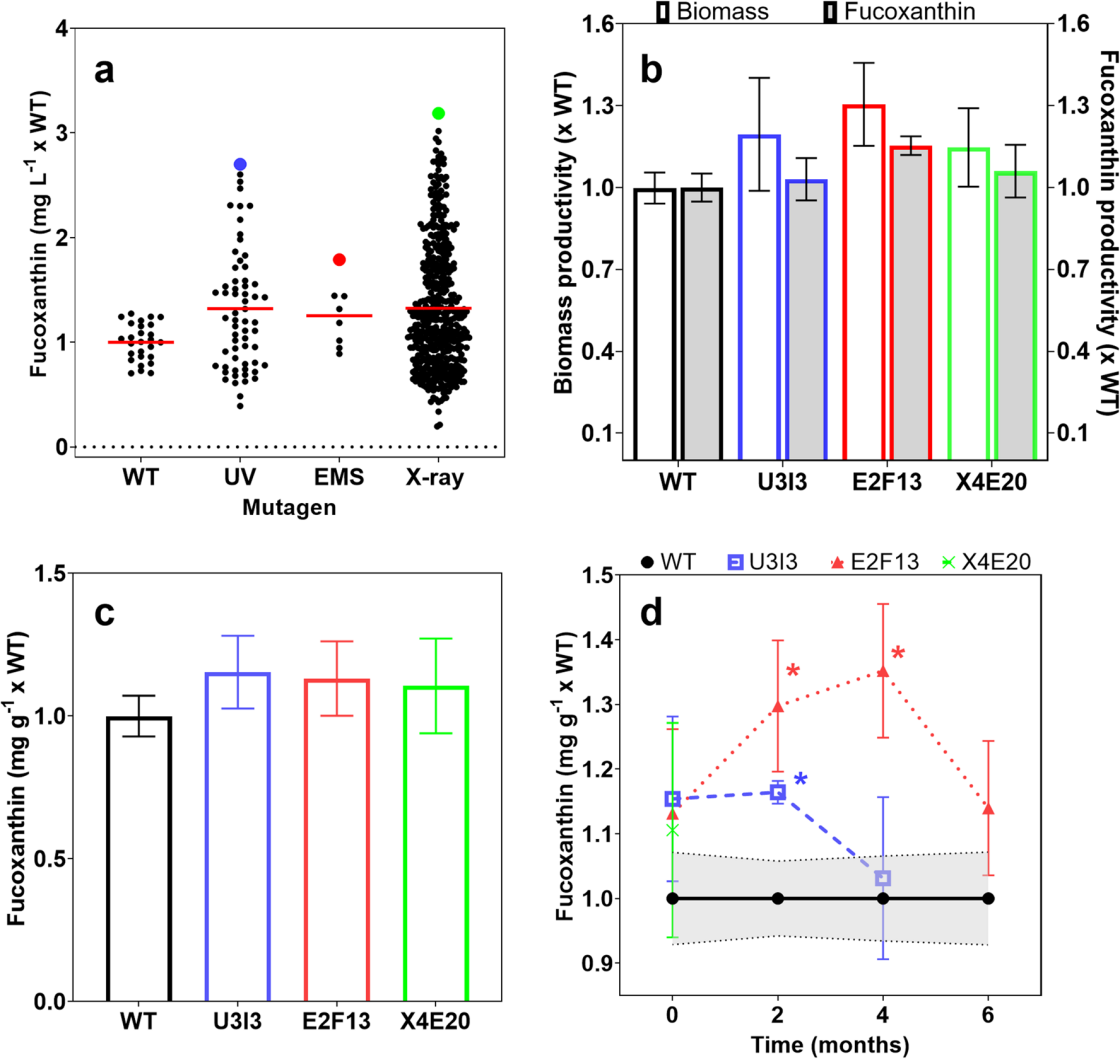
Spectral deconvolution, biomass and fucoxanthin data (**a**) results of spectral deconvolution screening method on WT, UV, EMS and X-ray treated populations (note that this is after FACS-sorting for higher fluorescence in mutagen-treated populations) with coloured dots indicating strains selected for further subculturing and culture and chemical analysis. Units are in mg L^−1^ as strains are measured in wells and the method accounts for OD as a function of biomass (OD is measured separately for information about growth), (**b**) Biomass productivity (mg dry biomass after lyophilisation per L per day) and fucoxanthin productivity results of top strain chosen from each mutagen, (**c**) fucoxanthin content (mg per g dry weight) of top strains, and (**d**) temporal analysis using HPLC over a 6-month period. U denotes UV treated strains, E denotes EMS treated strains and X denotes X-ray treated strains with asterisks denoting statistical significance with one-way ANOVA, *n* = 3 for month 0, *n* = 5 for months 2, 4 and 6.

The recovery of FACS-sorted single cells in 384-well plates is related to the mortality of a given treatment and was calculated by dividing the number of wells exhibiting at least some colour visible to the naked eye by the number of wells that had a single cell sorted into them. For example, UV showed a 76% mortality at day 7 and a 96% cell mortality after single-cell sorting while EMS had a 97% mortality and 99.7% survival after sorting. These are both contrasted to X-ray, which showed a 60% mortality and 11% survival after single-cell sorting. After cell recovery and screening, cells were chosen based on high OD and high fucoxanthin content as indicated using the spectral deconvolution method (Fig. 4a). The top performer from each mutagen were selected based on the highest fucoxanthin (mg L^−1^) compared to WT and subcultured in tissue culture flasks to measure culture characteristics and chemical analysis of pigment content. The sample ID for each was U3I3 (UV), E2F13 (EMS) and X4E20 (X-ray).

These top performers were between 200 and 300% of WT (strain mean fluorescence divided by WT mean fluorescence) fucoxanthin when measured using spectral deconvolution (Fig. 4a), yet these strains were between 111 and 115% of WT fucoxanthin when measured on HPLC several weeks later (Fig. 4c). This difference could be due to multiple factors including exaggeration of pigment results from the spectral deconvolution method, reduction in cellular pigment content after screening and differences in shading effects from growth flask formats. As a result, no strain was significantly higher than WT at time 0 (One-way ANOVA with p-value threshold < 0.05). However, one strain (U5E8) showed significantly higher volumetric biomass productivity (121% WT, p = 0.0302, data not shown). All 3 chosen strains displayed a higher mean volumetric biomass productivity than WT with E2F13 having 131% (SD = 15%) WT, although none were statistically significant (Fig. 3b). Neither U3I3 nor X4E20 had over 110% WT fucoxanthin productivity where E2F13 which had 115% (SD = 15%) WT volumetric fucoxanthin productivity (Fig. 4b).

Spectral deconvolution results showed a range of fucoxanthin from near 0 to over 300% WT even after sorting top cells using FACS with multiple weeks of incubation. Also, the standard deviation was larger in mutant populations (13 – 17%) compared to WT (7%) even after a further month of cultivation and measurement using HPLC, and it was presumed that strains were still undergoing genome instability^36^.

The top UV-C generated strain (U3I3) was stable for up to 2 months (${\overline{\text{x}}}$ = 115%, SD = 13% WT for month 0 and ${\overline{\text{x}}}$ = 116%, SD = 2% WT for month 2), before dropping for month 4 (${\overline{\text{x}}}$ = 103%, SD = 13% WT). The top EMS strain (E2F13) increased from 113% WT with a SD of 13% at month 0 to 130%, 135% and 114% WT at months 2, 4 and 6, respectively (all with SD = 10%). The top X-ray strain (X4E20) displayed 111% WT with a SD of 17% at month 0 (Fig. 4d). Due to fluctuations in fucoxanthin content, E2F13 was significantly higher than WT at months 2 and 4, while U3I3 was significantly higher at month 2 (one-way ANOVA, significance level 0.05, Fig. 4d).

## Discussion

### Mutagen effects on mortality and fluorescence

While mortality is a useful statistic for determining mutagen effects on cell cultures, combined mortality (Fig. 3a–c) and chlorophyll *a* fluorescence data (Fig. 3d) offers a complete view of populations generated by different mutagens. Chlorophyll *a* fluorescence is a simple measurement of photosynthetic health and serves to provide a reliable preliminary assessment of the effects of a given mutagen over time (Fig. 3d). Differences between mortality and chlorophyll fluorescence kinetics indicate that cells do not respond consistently to X-rays like they do with the non-ionising mutagens. While X-rays appear to either kill cells or not to have a significant effect on them as indicated by the split fluorescence populations (Fig. 3d), UV and EMS show a uniform loss of fluorescence throughout the 7 days after treatment. While this could be dose-dependent, the comparable mortality between X-rays and UV treatments indicates that cultures respond unevenly to X-ray exposure. In addition, Myung and Kolodner^37^ found that 0.7% EMS exposure for 2 h had a tenfold higher GCR (Gross Chromosomal Rearrangement) induction than 100 Gy gamma radiation in *Saccharomyces cerevisiae*. A combination of factors are responsible for the inability to truly compare effects between mutagens—differences in mortality at day 7 after treatment, the difference in peak shapes of chlorophyll fluorescence indicating uneven effects in X-ray cultures as well as the nature of the resulting mutations from each mutagen. EMS may simply be a more efficient tool for creating stable mutants, and this is reflected in EMS being used in 43% of reports where researchers used random mutagenesis to improve microalgae performance^38^. This could be due to EMS providing doses on shorter timeframes, for example EMS was used here to treat cells for 30 min, whereas 1000 Gy X-ray was delivered at 157 mGy/s for 1 h 46 min. It is important to note here that no statistical comparison between the mutagens is included here as their effects on cells are expected to differ considerably, rendering comparison between them difficult. Additionally, treatments displayed dissimilar mortality statistics, further making the task of comparison untenable. Therefore in this work all comparisons are merely observational. Other consideration are also important, such as the ease and cost of different methods. X-radiation should be incorporated as a mutagen in further research, with consideration for its lengthier and potentially cost-heavy involvement comparative to other methods.

While there was no intraspecific comparison of single-cell survivability between treatment intensities herein, treatments that result in higher mortality were observed to increase the proportion of surviving cells with mutations. Thus increasing the likelihood of finding a mutant with the desired trait in downstream analysis, despite the lower total cells screened. This can be seen in the screening of 8 EMS strains with 1 displaying significantly higher fucoxanthin in the temporal analysis and 62 UV strains being screened with 1 displaying significantly higher fucoxanthin in the temporal analysis whereas 445 X-ray strains were screened without any showing significantly higher fucoxanthin. This topic remains purely discussion in this work as there are no comparative measurements of UV-C radiation and X-radiation, nor between these and EMS, on cellular functioning and response. It is, however important to note that, in the process of creating mutants, there exists a balance between obtaining high mortality to maximise the possibility of mutations in surviving cells and preventing the elimination of the culture completely. Varying species are expected to respond differently to mutagens and recovery conditions, and therefore the optimal mortality range to maximise stable mutations could differ, despite a general understanding that 90–95% mortality is optimal. There is also the consideration that researchers should take care when deciding on a recovery point after treatment, considering the additional balance between the goal of filtering out undesired strains while simultaneously avoiding wasting screening and selection effort on the same strain multiple times, or ‘doubling down’.

### Sorting and screening

Fluorescence-Activated Cell Sorting is being widely recognised as a pivotal tool for the artificial selection of microbial cells, and indeed has been used to select for superior pigment-producing strains of microalgae^39–41^. The inclusion of FACS in this work also shows the immense utility of this tool as a means of sorting single cells, and therefore potentially distinct genomes, into individual wells for further culturing and analysis. Another vital function of FACS is to provide a relatively simple means of artificial selection for a target phenotype, and restricts the pool of mutant cells to a much higher threshold for that target phenotype. This is evident here in the increase of average spectral deconvolution results for fucoxanthin to 125–133% WT in mutant populations that have undergone single-cell sorting with FACS with an appropriate channel.

Including a secondary high-throughput screen in the form of spectral deconvolution allowed for sterile, non-invasive mathematical assessment of both culture density and a secondary filtering of strains based on fucoxanthin content. This was essential when considering 34% of cells screened using spectral deconvolution were lower than WT, further suggesting phenotype instability caused reversion to WT fucoxanthin contents between FACS-sorting and spectral deconvolution screening stages of the experiment. Choosing wells with low OD and high fucoxanthin predictions after spectral deconvolution is likely to select for higher fucoxanthin content regardless of biomass-related productivity, while selecting for high OD is likely to select for productivity. By comparison, selecting for high OD with high relative fucoxanthin is optimal but difficult to assess when WT wells used for comparison are of a similar density. It is unclear whether just genome instability or exaggeration of fucoxanthin in spectral deconvolution is the cause of high variance in spectral deconvolution results, but it is likely a combination of the two. The spectral deconvolution method here enabled the successful selection of 2 strains with significantly higher fucoxanthin than WT when assessed over 6 months, and the combination of FACS with spectral deconvolution screening in 384-well microplates enabled straightforward and effective isolation of strains from a vast pool of mutated cells (near 5000 cells sorted). Considering the complexity of carotenoid biosynthesis across genes and related regulatory mechanisms, the likelihood of creating a strain with improved carotenoid production is small. This work highlights the need to ensure high mortality in treatments to increase the proportion of DNA in a culture being affected by a given mutagen, as well as the importance of researchers maximising the quantity of cells screened after treatment. There is also potential to expand beyond single-event mutagenesis and to incorporate other strategies such as iterative mutagenesis and adaptive laboratory evolution.

### High performance liquid chromatography

The HPLC results for the temporal analysis supported previous indications that the top strain pigment phenotypes were unstable, and in fact during subculturing, we observed both decrease and increase in the average pigment content despite maintaining constant growth and sampling regimes over a 6-month period, which indicates the cultures were still highly heterogenous. Bulankova et al.^36^ investigated the nature of clonal variability in *P. tricornutum* haplotypes and found that mitotic interhomolog recombination rates were in excess of 10× that of *Saccharomyces cerevisiae*, indicating that genomic diversity is rapidly accumulated in *P. tricornutum* clonal cultures. It is probable that cells with higher pigment expression outcompeted others, and may have been outcompeted themselves later in the growth cycle. Mitotic recombination was found to increase under oxidative stress in *P. tricornutum*^36^, and reversion to wild-type pigment expression levels may also have been caused by eventual loss of a response to stress conditions brought on by exposure to mutagens. Secondary effects such as interactions with Reactive Oxygen Species (ROS) are also responsible for damage to DNA, and differences between the levels of direct mutations and secondary effects, as well as activity of DNA repair mechanisms, might explain the differing success with isolation of elite phenotypes between different mutagens^38,42,43^. Note that temporal measurements are from a single population inoculated for each 2-month HPLC cycle and not replicate flasks separately subcultured. This suggests ongoing sorting and screening, and possibly even mutagenesis, is essential for maintaining successful hyper-performing cultures over long periods of time.

This work proposes genome instability as the cause of high error in HPLC measurements of fucoxanthin and as the reason for fluctuating fucoxanthin content over the 6 month experimental period. Future studies are however needed to assess the exact cause of fluctuating population dynamics as a suspected result of mutation at the single-cell level. Additional measurements of ROS damage as secondary damaging effects as well as SOS repair would aid in this assessment as genomic stability after DNA damage is dependent on cellular repair mechanisms^35,44^.

## Conclusion

Both short timeframe (days to weeks) and long timeframe (months) reversion of selected strains to WT pigment levels suggest that ongoing laboratory evolution would be preferable for creating novel strains. Sustained selection pressure and artificial selection using FACS are both likely to encourage the continued expression of desired phenotypes. In addition, X-ray mutagenesis should be further investigated alongside laboratory evolution methods by the increase of treatment intensity as well as cell-level investigation of the dynamic effects of mutations in cultures. In the future, a more detailed comparison of ionising and non-ionising mutagens will reveal the benefits and disadvantages of utilising additional mutagenic mechanisms in evolution experiments.

## Data Availability

The datasets used and/or analysed during the current study are available from the corresponding author on reasonable request.
